# Outcomes of Drug-Coated Balloon Versus Drug-Eluting Stent for the Treatment of Large *De Novo* Coronary Lesions

**DOI:** 10.31083/RCM50502

**Published:** 2026-10-12

**Authors:** Wenjie Wang, Hongfei Si, Lingjuan Li, Xiliang Zhao, Quan Li, Yong Zeng, Xianpeng Yu

**Affiliations:** ^1^Department of Cardiology, Beijing AnZhen Hospital, Capital Medical University, 100069 Beijing, China; ^2^Department of Cardiology, Dong’e People’s Hospital, 252299 Liaocheng, Shandong, China; ^3^Department of Cardiology, Langfang Fourth People’s Hospital, 065700 Langfang, Hebei, China

**Keywords:** drug-coated balloon, *de novo* lesion, coronary artery disease, diagnosis

## Abstract

**Background:**

Although drug-coated balloons (DCBs) outperform drug-eluting stents (DESs) in the treatment of *de novo* small-vessel coronary lesions, no comparable studies are available for *de novo* large-vessel coronary lesions.

**Methods:**

This retrospective study examined 960 patients with *de novo* large-vessel coronary lesions (reference vessel diameter ≥3.0 mm) who underwent paclitaxel DCB angioplasty or DES implantation at Beijing Anzhen Hospital. The study endpoints included all-cause death, myocardial infarction (MI), stroke, and target vessel revascularization (TVR). Kaplan–Meier (KM) curves and Cox regression analysis were used to investigate the clinical outcomes of patients in the DCB and DES groups.

**Results:**

The overall incidence rates of major adverse cardiac and cerebrovascular events (MACCEs), all-cause death, MI, stroke, and TVR were 9.27% (89/960), 1.5% (14 patients), 0.9% (9 patients), 0.6% (6 patients), and 6.8% (65 patients), respectively. No significant differences were observed between the DCB and DES groups in MACCEs (13.2% vs. 5.6%; *p* = 0.511), all-cause death (1.9% vs. 1.0%; *p* = 0.763), MI (0.6% vs. 1.2%; *p* = 0.239), or stroke (0.6% vs. 0.6%; *p* = 0.361). However, the TVR rate was significantly higher in the DCB group than in the DES group (10.1% vs. 3.6%; *p* = 0.028), and this difference persisted after multivariate adjustment.

**Conclusions:**

For patients with *de novo* lesions in target vessels with diameters ≥3.0 mm, the safety and efficacy of DCB therapy were comparable to those of DES therapy, except for target vessel revascularization.

## 1. Introduction

Percutaneous coronary intervention (PCI) utilizing drug-eluting stent (DES) implantation is the well-established gold standard for the nonpharmacological management of symptomatic coronary artery disease (CAD). A major limitation of metallic stents is their permanent implantation as foreign bodies, which fosters a proinflammatory environment and may cause hypersensitivity, neoatherosclerosis, and eventual stent thrombosis [1,2]. The use of drug-coated balloons (DCBs)—semicompliant balloons that dilate stenotic coronary lesions without leaving metallic residues—reduces the rate of restenosis and has therefore drawn considerable attention as an “intervention without implantation” strategy [3]. DCB therapy can potentially attenuate long-term stent-related adverse events and alleviate dual antiplatelet therapy–associated bleeding risk in patients with small-vessel CAD, thereby offering an advantage over DES implantation [4,5]. The BASKET-SMALL 2 trial [6] showed that the efficacy of DCB therapy for *de novo* small-vessel CAD is noninferior to that of DES therapy, with no significant difference in the incidence rates of three-year major adverse cardiovascular events (MACEs) observed between the two groups. In contrast, the PICCOLETO II trial indicated that DCB therapy is superior to DES therapy in terms of late lumen loss in small-vessel CAD [7].

The role of DCB therapy in treating large-vessel disease (LVD), i.e., *de novo* lesions in large coronary vessels (diameter ≥3.0 mm), is inconsistent yet potentially promising. This therapy offers the unique advantage of avoiding stent strut malposition, particularly in vessels with irregular walls, aneurysmal dilation, or bifurcation anatomy. To clarify the clinical value of DCB-based LVD therapy, this study examines its long-term clinical outcomes using real-world data.

## 2. Materials and Methods

### 2.1 Patients and Procedure

This retrospective study involved 960 patients with LVD who underwent paclitaxel DCB angioplasty (462 patients) or DES implantation (498 patients) at Beijing Anzhen Hospital from 1 June, 2019, to 30 November, 2021. Large vessel disease (LVD) was characterized by a reference vessel diameter (RD) of ≥3.0 mm. The DCB utilized in the present study was the SeQuent Please device (B. Braun Melsungen AG, Melsungen, Germany), which adopts a butyl methacrylate copolymer matrix and delivers paclitaxel at a dose of 3 μg/mm^2^. All implanted drug-eluting stents (DES) were contemporary mainstream new-generation devices.

All patients received either DCB alone or DES alone; patients who underwent combined treatment with both DCB and stents were excluded from the study.

Prior to the use of the paclitaxel-coated balloon catheter, adequate predilation was performed with a balloon-to-vessel diameter ratio maintained at 0.8–1.0. Non-compliant (NC) balloons, cutting balloons, scoring balloons, or non-slip element (NSE) balloons were used when necessary. Patients were deemed eligible for DCB therapy if predilation afforded a satisfactory result (residual stenosis rate ≤30%, TIMI grade 3 flow, and no dissection or only type A/B dissection of the target lesion). Both DCB ends were positioned 2–3 mm beyond the lesion margins, and inflation was maintained at 8–10 atm for a minimum of 30 s. The procedure was considered successful if a residual stenosis rate of ≤30% and TIMI grade 3 flow were achieved. DCB treatment was considered unsuccessful if a notable dissection (NHLBI type C or higher) was present alongside TIMI flow of grade <3, at which point bailout stenting with DES was implemented. Patients who underwent bailout stenting with DES were excluded from the final analysis, i.e., only those with successful DCB intervention were included.

All participants provided written informed consent for the procedure and subsequent use of their clinical data for research purposes. The study was conducted in accordance with the Declaration of Helsinki and approved by the local ethics committee (Beijing Anzhen Hospital Ethics Committee of Capital Medical University), which additionally authorized the utilization of clinical data for the present study. No industry actors were involved in the study design, conduct, funding, or data analysis.

### 2.2 Clinical Definitions and Follow Up

Clinical follow-up was conducted at 1 month, 6 months, 1 year, and annually thereafter until the occurrence of one of four endpoints: all-cause mortality, myocardial infarction, stroke, and target vessel revascularization. Follow-up data were collected through outpatient visits, telephone interviews, and coronary angiography. For patients presenting with ischemic symptoms or signs during the follow-up period, angiographic reexamination was recommended but not mandatory. All clinical endpoints were confirmed by source documentation and adjudicated by the local clinical event committee of Beijing Anzhen Hospital, Capital Medical University.

MI was identified based on evidence of myocardial necrosis in alignment with the Third Universal Definition of Myocardial Infarction [8]. Stroke was confirmed by a neurologist using imaging studies to verify neurological impairment. MACCE was defined as a composite endpoint of the first occurrence of all-cause death, stroke, MI, or TVR. All outcomes were initially identified by the patients’ attending physicians and subsequently subjected to central adjudication by an independent clinical committee at Beijing Anzhen Hospital. In the cumulative analysis, events were treated as single occurrences, with priority given to the earliest event.

### 2.3 Statistical Analysis

Continuous data were expressed as either means ± standard deviations (SD) or medians with interquartile ranges (IQR). Comparisons between groups were performed using the independent samples Student’s *t*-test or the Mann-Whitney U test, depending on the normality of the data distribution. Categorical variables were reported as counts and percentages, and differences between the two groups were assessed by means of the chi-square test. The time to the primary endpoint was estimated using the Kaplan–Meier method, and the log‑rank test was employed to compare endpoint incidence between the LVD and small-vessel disease (SVD, lesions with a reference vessel diameter of less than 2.75–3.0 mm) groups. Cox proportional hazards regression analyses were performed to adjust for confounding factors, including age, sex, diabetes mellitus, smoking, hypertension, dyslipidemia, prior stroke, prior myocardial infarction (MI), prior coronary artery bypass grafting (CABG), and prior percutaneous coronary intervention (PCI). All statistical analyses were conducted using SPSS software (Version 22.0, IBM Corp., Armonk, NY, USA) for Windows. A two‑sided *p* value < 0.05 was deemed statistically significant.

## 3. Results

A total of 960 patients who underwent paclitaxel drug-coated balloon (DCB) therapy or drug-eluting stent (DES) implantation for *de novo* coronary artery lesions between June 1, 2019, and November 30, 2021, were finally enrolled in this retrospective analysis. Among them, 462 patients received DCB treatment (DCB group) and 498 patients underwent DES implantation (DES group).

The baseline clinical characteristics of the two groups are detailed in Table 1. The results showed that the incidences of smoking history, diabetes mellitus, hyperlipidemia, prior coronary artery bypass grafting (CABG), and percutaneous coronary intervention (PCI) were significantly higher in the DCB group than in the DES group. No statistically significant differences were observed between the two groups in terms of age, gender distribution, hypertension history, stroke history, clinical phenotypes, and the number of diseased vessels (all *p* > 0.05).

**Table 1.** **Baseline clinical characteristics**.

Variables		DCB group (n = 462)	DES group (n = 498)	*p* value
Age, years		58 (50–66)	57 (51–67)	0.062
Male (%)		384 (83.1)	410 (82.3)	0.747
Diabetes mellitus (%)		162 (35.1)	125 (25.1)	0.001
Smoking history (%)		286 (61.9)	213 (42.8)	<0.001
Hypertension (%)		267 (57.8)	268 (53.8)	0.215
Dyslipidemia (%)		265 (57.4)	79 (15.9)	<0.001
Prior stroke (%)		30 (6.5)	32 (6.4)	0.966
Prior MI (%)		101 (21.9)	174 (34.9)	<0.001
*Prior CABG		13 (2.8)	0 (0)	<0.001
Prior PCI (%)		91 (19.7)	0 (0)	<0.001
eGFR		96.4 (88.4–103.8)	96.3 (87.3–102.9)	0.431
LVEF, %		64 (60–66)	63 (57–68)	0.501
sCr, μmol/L		73.7 (64.8–82.5)	73.4 (64.7–84.1)	0.629
Clinical presentation (%)				0.539
	NSTEMI	21 (4.5)	26 (5.2)	
	STEMI	18 (3.9)	22 (4.4)	
	Stable angina	52 (11.2)	60 (12.0)	
	Unstable angina	371 (80.3)	390 (78.3)	
Extent of diseased vessel (%)				0.490
	LM	21 (4.5)	24 (4.8)	
	single-vessel disease	219 (47.4)	225 (45.2)	
	double-vessel disease	136 (29.4)	141 (28.3)	
	triple-vessel disease	86 (18.6)	108 (21.7)	

PCI, percutaneous coronary intervention; CABG, coronary artery bypass grafting; PVD, peripheral vascular disease; LVEF, left ventricular ejection fraction; NSTEMI, non-ST-elevation myocardial infarction; STEMI, ST-elevation myocardial infarction; LM, left main. *Fisher’s exact test was used for the comparison of Prior CABG.

The baseline lesion characteristics are presented in Table 2. The proportions of left anterior descending artery and type B2/C lesions were significantly higher in the DCB group, whereas those of left circumflex artery, right coronary artery, and bifurcation lesions were significantly higher in the DES group. No significant differences were observed between the two groups in terms of the incidences of left main coronary artery, ramus intermedius, chronic total occlusion, and calcified lesions, as well as in reference vessel diameter and lesion length (all *p* > 0.05).

**Table 2.** **Baseline lesion characteristics**.

Variables		DCB group (n = 515)	DES group (n = 549)	*p* value
Lesion location				
	LAD/D	248 (48.2)	254 (46.2)	<0.001
	LCX/OM (%)	80 (15.5)	91 (16.6)	<0.001
	RCA/PDA/PL (%)	176 (34.2)	193 (35.2)	<0.001
	LM (%)	9 (1.7)	8 (1.5)	0.688
Ramus intermedius (%)		2 (0.4)	3 (0.5)	0.715
CTO (%)		19 (3.7)	32 (5.8)	0.110
Calcified lesions (%)		22 (4.3)	33 (6.0)	0.214
Bifurcation (%)		131 (25.4)	152 (27.6)	<0.001
B2/C lesions (%)		333 (64.7)	342 (62.2)	<0.001
Reference diameter (mm)		3.0 (3.0–3.5)	3.0 (3.0–3.5)	0.627
Lesion length (mm)		22.5 ± 7.2	22.9 ± 7.8	0.459

The median follow-up duration for all patients was 31.6 months (interquartile range, IQR: 19.4–37.4 months). During the follow-up period, a total of 89 MACCEs (incidence rate: 9.27%), 14 all-cause deaths (incidence rate: 1.5%), 9 myocardial infarctions (MIs) (incidence rate: 0.9%), 6 strokes (incidence rate: 0.6%), and 65 target vessel revascularizations (TVRs) (incidence rate: 6.8%) occurred among the 960 patients. Kaplan–Meier (KM) survival curve analysis (Fig. 1) revealed no statistically significant differences in the cumulative incidences of all-cause death, MI, and stroke between the DES and DCB groups (all *p* > 0.05). However, the risk of repeat revascularization was significantly lower in the DES group than in the DCB group.

**Fig. 1.**
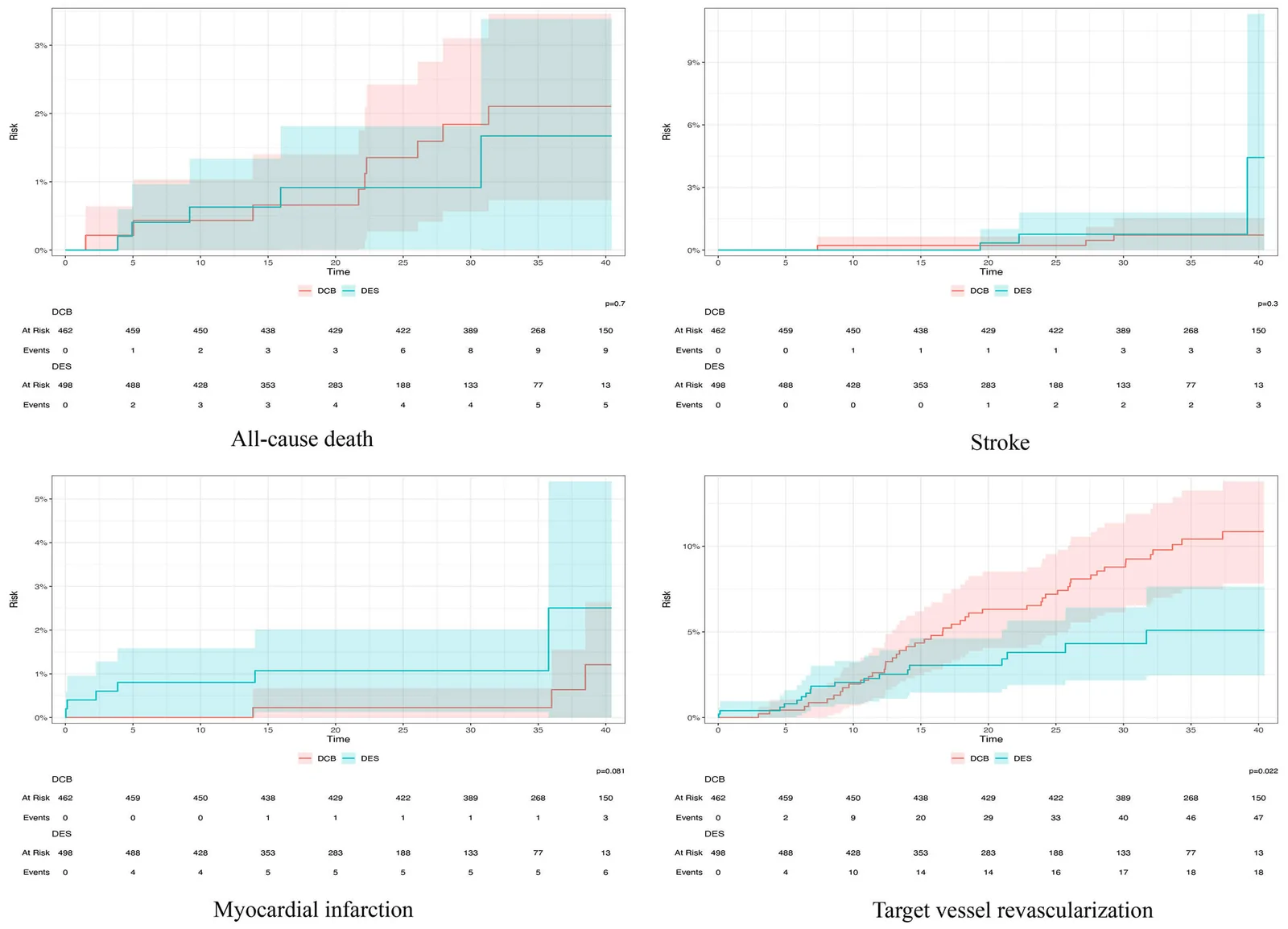
**Kaplan‑Meier curves for clinical outcomes comparing drug‑coated balloon (DCB) versus drug‑eluting stent (DES) treatment**.

Multivariate adjusted Cox regression analysis demonstrated that compared with the DCB group, the DES group had a significantly reduced risk of TVR [hazard ratio (HR) = 0.538, 95% confidence interval (CI): 0.309–0.937, *p* = 0.028]. No significant differences were found between the two groups in the risks of other adverse outcomes, including all-cause death, stroke, and MI (all *p* > 0.05) in Table 3.

**Table 3.** **Clinical outcomes**.

Outcomes		DCB	DES	*p* value	HR (95% CI)
Unadjusted					
	Death	1.9%	1.0%	0.724	0.818 (0.268–2.496)
	MI	0.6%	1.2%	0.081	3.483 (0.802–15.127)
	Stroke	0.6%	0.6%	0.346	2.185 (0.415–11.514)
	TVR	10.1%	3.6%	0.022	0.530 (0.306–0.921)
	MACCE	13.2%	5.6%	0.094	0.677 (0.429–1.069)
Adjusted					
	Death	1.9%	1.0%	0.763	0.842 (0.276–2.568)
	MI	0.6%	1.2%	0.239	2.413 (0.556–10.465)
	Stroke	0.6%	0.6%	0.361	2.163 (0.413–11.334)
	TVR	10.1%	3.6%	0.028	0.538 (0.309–0.937)
	MACCE	13.2%	5.6%	0.511	0.842 (0.505–1.404)

HR, hazard ratio; MI, myocardial infarction; TVR, target vessel revascularization.

## 4. Discussion

This investigation evaluated the safety profiles of patients with *de novo* large-vessel coronary artery disease undergoing treatment with either drug-coated balloon (DCB) angioplasty or drug-eluting stent (DES) implantation. Our findings revealed that while DES therapy was associated with a significantly lower rate of target vessel revascularization (TVR) compared to DCB, the incidence of all-cause death, myocardial infarction (MI), and stroke did not differ significantly between the two cohorts.

DCB angioplasty is primarily indicated for in-stent restenosis [9], although a growing body of evidence supports its application in the treatment of *de novo* coronary artery lesions, and it is expected to become a novel therapeutic strategy for SVD. The RESTORE SVD China trial—the first multicenter randomized controlled trial (RCT) in China comparing DCB therapy with a new-generation DES therapy—enrolled 230 patients with SVD who were randomly assigned to DCB and DES groups at a 1:1 ratio. Angiographic follow-up at 9 months revealed no significant difference in the percentage of diameter stenosis within the target segment between the two groups [10]. A retrospective study from Beijing Hospital, China, showed that the efficacy and safety of paclitaxel DCB therapy (SeQuent Please®) for *de novo* LVD (reference vessel diameter ≥2.8 mm) were comparable with those for SVD (reference vessel diameter <2.8 mm) [11]. Additionally, the BASKET-SMALL 2 trial demonstrated that paclitaxel DCB therapy was noninferior to second-generation DES therapy in terms of the composite endpoint of cardiac death, nonfatal MI, or TVR at 12 months post procedure [12]. The DEBUT trial enrolled 208 patients with LVD (reference vessel diameter = 2.5–4.0 mm) and randomly assigned them to DCB and DES groups. The efficacy of DCB angioplasty was superior to that of bare-metal stent (BMS) implantation [13]. In another study enrolling patients with left main bifurcation lesions following directional coronary atherectomy, the DCB group was associated with a higher TLR rate, whereas the incidence of myocardial infarction was comparable between groups [14]. In the present study, we compared stent implantation with DCB angioplasty in large-vessel lesions and found no statistically significant differences in the incidences of all-cause death, MI, or stroke between the two groups.

The REVELATION trial evaluated fractional flow reserve at 9 months postprocedure in patients with acute MI and demonstrated that DCB therapy was noninferior to DES therapy, suggesting that the former may be a safe and feasible option for such patients [15]. The PEPCAD non-ST-segment elevation myocardial infarction (NSTEMI) trial primarily enrolled patients with NSTEMI who underwent DCB therapy for *de novo* coronary artery lesions, showing that the efficacy of this therapy was noninferior to that of BMS or DES implantation [16]. Another meta-analysis including 10 RCTs found no significant difference in safety between DCB and DES groups, although the rate of target lesion revascularization (TLR) in the DCB group was lower than that in the BMS group. In patients with ST-segment elevation myocardial infarction (STEMI), no significant differences in safety outcomes or late lumen loss were observed between DCB and DES groups, although the minimal lumen diameter was smaller in the DCB group. For patients excluding those with STEMI, the DCB group had lower MACE and TLR incidences than the overall stent group [17]. In another meta-analysis that included patients with a reference vessel diameter of ≥2.5 mm, the DES group showed superior immediate vessel expansion. However, there was no statistically significant difference in long-term LLL compared with the DCB group, suggesting comparable long-term luminal preservation between the two treatment strategies [18]. The REC-CAGEFREE I study, which included 2272 patients with *de novo* noncomplex coronary lesions (without restriction on target vessel diameter) and followed them up for 3 years, found that the incidence of device-oriented composite endpoint within 3 years was higher in the DCB group than in the stent group. This finding was mainly attributed to an increased rate of repeat revascularization. However, no significant differences in the incidence of cardiac death or target vessel MI were observed between the two groups [19]. In our study, the rate of target lesion revascularization was higher in the DCB group compared with the stent group, which represents a consistent finding across this real-world retrospective analysis. Notably, the DCB group harbored a higher baseline burden of patients with diabetes mellitus, hyperlipidemia, smoking history, and prior percutaneous coronary intervention. Although rigorous multivariate adjustment was performed to balance these confounders, such baseline imbalances may still have influenced the long‑term risk of subsequent revascularization. Further adequately powered randomized controlled trials are therefore warranted to clarify these observations and validate the comparative clinical outcomes.

A meta-analysis including 14 RCTs with a total of 2483 patients (1268 in the DCB group and 1215 in the control group) revealed that the overall clinical outcomes of DCB therapy for *de novo* coronary artery lesions were comparable with those of DES therapy, BMS therapy, or plain old balloon angioplasty. Furthermore, DCB therapy exhibited advantages in terms of late lumen loss, all-cause mortality, MI risk, and particularly reduced TLR compared with BMS [9]. A study from Japan enrolled patients with acute coronary syndrome (ACS) and single-vessel disease who underwent DCB therapy or DES implantation. After 1-year follow-up, the clinical outcomes of DCB therapy were similar to those of DES implantation in successfully treated patients, with the two therapies showing comparable efficacy and safety regardless of lesion location or ACS subtype [20]. A study comparing the safety and efficacy of sirolimus-coated balloon (SCB) therapy with those of second-generation DES therapy in PCI for patients with CAD aged ≥75 years found that the two groups showed comparable 2-year risks of net adverse clinical events and ischemia-related adverse events and no differences in safety or efficacy. SCB therapy significantly reduced the risk of major bleeding and demonstrated superior MACE and bleeding control effects in the ISR subgroup, an advantage that may be associated with the shorter duration of dual antiplatelet therapy in the SCB group [21]. The above studies suggest that DCB angioplasty is a promising therapeutic modality for the future.

DCB angioplasty is associated with a relatively high risk of post-procedure coronary artery dissection. Zhang et al. [22] reported that the incidence of coronary artery dissection in the DCB group significantly exceeded that in the DES group (8.3% vs. 3.0%, *p* = 0.02). Compared with the DES group, the DCB group showed higher incidences of in-hospital recurrent MI, no-reflow phenomenon, and TLR, most of which were associated with delayed coronary artery dissection. In their prospective observational study, Cortese et al. [23] found that one-third of patients developed dissection after DCB angioplasty; however, all dissections healed completely on follow-up angiography and did not increase the incidence of adverse cardiovascular events. Hui et al. [24] analyzed the impact of post-DCB dissection on the angiographic and clinical outcomes of coronary lesion treatment. Herein, we primarily focused on the risk of dissection in large-vessel lesions, as this risk is more noteworthy. After long-term follow-up, no significant differences in the risks of postprocedure MI, stroke, or all-cause death were observed between the two groups. However, given that dissection occurs after DCB angioplasty in large vessels, operators may be more inclined to implant stents to achieve higher procedural comfort and safety. The number of cases where DCB treatment was abandoned and replaced with stent implantation because of significant elastic recoil or flow-limiting dissection after predilation is unknown, and so is the number of cases when bailout stenting was implemented because of severe dissection. As we aimed to evaluate the efficacy and safety of DCB-only and DES-only strategies, patients who underwent bailout stenting were excluded.

## 5. Limitation

This study has several limitations. First, it is a single-center retrospective registry study. Although the data were derived from a large cardiac center, the inclusion of multicenter clinical data would further validate the results. Second, the follow-up duration after DCB treatment is relatively short and may be extended in future studies. Third, given the retrospective nature of this study, bailout stenting cases could not be reliably ascertained and the utilization rate of intravascular imaging (IVUS/OCT) was imbalanced between the DCB and DES groups. Such discrepancy may affect lesion preparation and procedural optimization, thereby introducing potential bias to the comparative outcomes.

## 6. Conclusion

In patients with *de novo* lesions in large vessels (reference diameter≥3.0 mm) subjected to DCB or DES therapy, safety and efficacy were comparable during midterm follow-up. However, the risk of TVR was significantly higher in the DCB group.

## Data Availability

The datasets generated and analyzed during the current study are available from the corresponding authors on reasonable request.
